# Black Women’s Experiences Along the HIV Care Continuum in the United States: A Scoping Review

**DOI:** 10.1089/heq.2024.0020

**Published:** 2024-09-12

**Authors:** Jacqueline P. Thomas, Will Ballew, Miu Ha Kwong

**Affiliations:** University of West Florida, Pensacola, Florida, USA.

**Keywords:** HIV, Black women, health disparities, health equity, HIV care

## Abstract

**Purpose:**

The prevalence of HIV among Black women is higher than the prevalence among other ethnic groups. Although antiretroviral therapy reduces HIV transmission and mortality, Black women still face health disparities when it comes to receiving health care. The purpose of this scoping review is to synthesize research regarding health disparities and health inequities faced by Black women living with HIV (BWLH).

**Methods:**

We searched three scholarly databases, PsychNet, MEDLINE, and CINAHL, and 18 peer-reviewed complete studies that met the inclusion criteria.

**Results:**

Several themes emerged from the literature, including discrimination, poverty, mental and physical health, health care, and social support. Each theme had a role in the progression of BWLH along the HIV care continuum.

**Conclusion:**

Black women continue to be disproportionately affected by HIV, which involves active engagement in HIV care to sustain viral suppression to prevent the spread of the virus. Factors continue to exist that contribute to health disparities and inequities, such as discrimination, internal and enacted HIV-related stigma, and poverty. Thematic findings in this review indicate that patient-centered care and support systems can positively impact BWLH experiences along the HIV continuum.

## Introduction

In the United States, 1.2 million people had HIV at the end of 2021.^1^ In total, 40% were Blacks/African Americans, representing 12% of the U.S. population. In contrast, Whites made up 61% of the U.S. population and 26% of HIV-positive people. Black women are disproportionately affected by HIV, with approximately 27% infected.^2^ There were 10 times as many new HIV infections among Black women compared to White women and four times as many cases compared to Latina women between 2015 and 2019.^2^ Most new HIV infections among females were caused by heterosexual contact. When transmitted, HIV-positive status increases the risk for new infections, which is a significant public health concern.^3,4^

Since its development in the 1980s, antiretroviral therapy (ART) has suppressed HIV viral loads, prevented HIV transmission, preserved health, and increased life expectancy.^5^ PrEP, a preexposure prophylaxis, and PEP, postexposure prophylaxis, are both additional HIV care advancements for people at risk for HIV due to sex or injection drug use.^6,7^ As a result of racism, HIV-related stigma, homophobia, and poverty, there are still disparities in equitable health care, explaining why HIV affects specific populations more than others.^6^ In terms of HIV-related stigma and discrimination, Black women with HIV face challenges impeding their chances to be linked to care or to maintain viral suppression, ultimately halting progression along the HIV care continuum.^6,8^ Access to HIV care and viral suppression are among the National Priorities to improve HIV-related health outcomes and diminish health disparities and health inequities.^4^ Therefore, the purpose of conducting this literature review is to gain a deeper understanding of HIV-related health disparities and health equity issues that affect Black women living with HIV (BWLH). Due to the disproportionate prevalence for Black women compared to other populations, it remains critical to understand how these disparities hinder access to and retention in HIV care, thus preventing viral suppression and increasing the risk for HIV transmission.

## Methods

The Arksey and O’Malley scoping review framework was used to identify empirical research articles published in peer-reviewed journals that explored the breadth of health disparities and health inequities experienced by BWLH.^9^

### Inclusion and exclusion criteria

The sample of articles collected for this scoping review included studies ranging from quantitative correlational research to qualitative studies that held several interviews with BWLH from local communities. All articles selected for this study qualified by (1) being written in English, (2) presenting original work, (3) being published in a peer-reviewed journal, (4) being published between January 2018 and July 2023, (5) containing samples of all female adult subjects, and (6) reporting data exclusively from subjects living in the United States. The aforementioned qualifications were applied across all databases; however, the ability to specify publication date ranges for articles within MEDLINE was limited to years.

Furthermore, articles satisfying the previous set of requirements were all individually reviewed to narrow down the sample of articles to only those that present information regarding health disparities and health inequities among BLWH. To be included, the articles in this phase had to satisfy the following criteria: (1) the studied sample must focus on HIV-infected or individuals living with HIV, (2) the sample should report at least 50% Black or African American women, and (3) health disparities or health inequities must be mentioned in the abstract. In addition, any articles remaining in the sample were excluded from the study if they included samples of individuals who were male, transgender, or not diagnosed or living with HIV.

### Search strategy

An initial literature search on August 14, 2023, spanned three scholarly databases: PsychNet, MEDLINE, and CINAHL. From the initial search, results included 160 articles from APA PsychNet, 350 articles from MEDLINE, and 106 articles from CINAHL. Two authors reviewed each article’s title and abstract within 4 weeks.

### Search terms

The authors used the same search terms to generate the initial number of articles across all three databases. First, to ensure articles highlighted BWLH populations, initial search terms included HIV, women, and health disparities. Furthermore, within each database, the “apply equivalent terms” setting was activated to identify articles with similar but different terms that were effectively equal to the input search terms. For example, an equivalent phrasing of “Black women living with HIV” would be “African American women diagnosed with HIV.”

### Study selection

To end the initial search, all authors scanned the titles, abstracts, and keywords of the remaining articles to confirm whether each article fit the inclusion and exclusion criteria. Next, citations were input into a storage folder within the online reference software Zotero. Moreover, articles were organized into one of three subfolders corresponding to the database the article was selected from. Additionally, any duplicate articles found were removed from the sample during this process. Afterward, the complete assortment was divided and screened in full text before deciding whether the article would be included in the review.

### Data extraction and organization

In addition to using Zotero, an Excel sheet was used to log the following information for each article: first author, year of publication, country of origin, keywords depicting main objectives of the study, type of research featured in the study, accessibility information including DOI numbers, sample percentages for BWLH, key findings, conclusions, and comments for how each article contributes to the research question for this literature review. When encoding the types of study, one of four labels was assigned to each article based on information provided in the study’s abstract and methods section. These labels included correlational, cross-sectional, longitudinal, and qualitative interviews. Additionally, information for each article was condensed and recorded in an Excel sheet (Table 1). The final information that was recorded included the first author, year of publication, type of research featured in the study, percentage of BWLH in the study sample, keywords, and key findings (Table 1). In addition, all data were thematically sorted into categories based on the disparities discussed.

**Table 1.** Characteristics of Included Studies

First author	Year published	Type of study/analysis	Location	Percentage of BWLH in sample	Objectives/keywords	Key findings
Budwhani, H.	2021	Correlational	Included participant data from: Brooklyn, NY Bronx, NY Chicago, IL Washington, DC Atlanta, GA Birmingham, AL Jackson, MS Miami, FL Chapel Hill, NC	82%	Experienced and anticipated stigma, patient–provider race concordance, missed appointments, distrust in health care providers	Higher experienced stigma was associated with lower trust in providers in all patient-provider race combinations
Cope, A.B.	2020	Correlational	Analysis completed in Chapel Hill, NC Included participant data from: Brooklyn, NY Bronx, NY Chicago, IL Washington, DC San Francisco, CA Atlanta, GA Birmingham, AL Jackson, MS Miami, FL Chapel Hill, NC	73%	Contextual poverty, viral suppression, blood pressure, health disparity	Neighborhood poverty was associated with unsuppressed viral load
Cressman, A.E.	2020	Correlational	Included participant data from: Included participant data from: Brooklyn, NY Bronx, NY Chicago, IL Washington, DC San Francisco, CA Atlanta, GA Birmingham, AL Jackson, MS Miami, FL Chapel Hill, NC	74%	Social discrimination, outpatient care, health status disparities, missed appointment	Women experiencing discrimination had a higher prevalence of missing HIV care appointments
Dale, S.K.	2018	Qualitative interviews	Boston, MA	100%	Resilience, HIV, social support, HIV stigma, trauma, racism, gender-related stressors	Social support and resilience are important protective factors for health disparities for BWLH
Dale, S.K.	2019	Longitudinal	Unnamed city in Southeast United States	100%	Microaggression, discrimination, mental health	Higher race-related discrimination, higher HIV-related discrimination and higher gendered racial microaggressions (GRM) significantly predicted higher total barriers to care
Dale, S.K.	2023	Longitudinal	Unnamed city in Southeast United States	100%	Microaggression, structural equation modeling, discrimination	Compared to non-Black women, Black women in the United States are more likely to be diagnosed with HIV, living with HIV, and have suboptimal HIV outcomes
Demeke, H.B.	2018	Cross-sectional	National data reported to the CDC	79%	Absolute and relative disparities, US-born, non-US-born, diagnosis rates	Differences in disparities in HIV diagnoses exist between U.S. and non-U.S. born Black women
Fabian, K.	2020	Correlational	Participant data from: 1. Chicago, IL 2. Birmingham, AL	100%	Internalized HIV-related stigma, breast cancer, social support, stigma reduction intervention	Internalized HIV-related stigma was associated with greater overall perceived threat, susceptibility, fear, and perceived adverse consequences of breast cancer
Koch, A.	2022	Qualitative interviews	Interviews collected from a sample of BWLWH in North Carolina	91%	Resilience, social support, coping, patterns of reciprocity	Coping and resilience themes among BWLWH include: self-acceptance, social support, disclosure, self-compassion, will-to-live, service
Levy, M.E.	2021	Correlational	Washington, DC	53%	Psychosocial stress, mental health, socio-structural vulnerabilities, cardiovascular disease	Psychosocial stress is a plausible mechanism for cardiovascular abnormalities over time for Black women
McMillian-Bohler, J.M.	2023	Qualitative interviews	Southern United States	86%	Stigma, HIV disclosure, Adaptive Leadership Framework for Chronic Illness	Challenges for BWLH include: Technical challenges: misconceptions about HIV and recognizing need for support Adaptive challenges: fear of rejection due to disclosure and lack of trust in family to keep diagnosis confidential
McCoy, K.	2020	Longitudinal	Chicago, IL and Birmingham, AL	100%	Engagement, HIV-related stigma, social support	Engagement in HIV-treatment was not found to be related to HIV-stigma but was found to be related to age, education level, and social support
Michel, K.G.	2022	Correlational	Washington, DC	77%	Trust, medication adherence, antiretroviral therapy, human immunodeficiency	Self-reported antiretroviral therapy adherence significantly associated with high provider trust, yet also with high healthcare system distrust, revealing a nuanced relationship to providers and the health care system among women with HIV
Molina, Y.	2018	Correlational	Washington, DC Participant data from: Chicago, IL Birmingham, AL	100%	HIV-related stigma, breast cancer, comorbidity, internalized stigma	Negative experiences with a particular diagnosis (HIV) may lead to greater negative appraisals of another diagnosis (breast cancer), in part because of internalization/self-rejection from the negative experiences of HIV
Nace, A.	2021	Correlational	Statesunder the Women of Color (WOC) Initiative enrolled WOC in the study from: 1. Alabama 2. California 3. Florida 4. Illinois 5. Massachusetts 6. North Carolina 7. New York 8. Texas	72%	Viral Suppression, Foreign-Born, U.S.-Born, education, housing, nativity	Foreign born HIV + WOC were more likely to achieve viral suppression than U.S. counterparts regardless of education and housing status
Ojukwu, E.	2022	Qualitative Interviews	South Florida, USA	100%	Geriatrics, health disparities, HIV treatment engagement, sociological factors	Positive influences for seeking treatment include: supportive social networks, perceived benefits, HIV knowledge, increased community awareness of HIV, impact of HIV legislation Barriers for seeking HIV treatment: low-income, substance use, HIV-related stigma, influence of stereotypes, health insurance, religion, comorbidities, HIV disclosure, and caregiving roles
Raiford, J.L.	2023	Correlational	Participant data collected from: 16 U.S. states Puerto Rico Washington DC Medical Monitoring Project (MMP) Sample	61%	Disparities, antiretroviral, adherence, viral suppression	Racial/Ethnic disparities in HIV outcomes among women taking ART were substantially reduced after accounting for social determinants of health
Watson, L.	2019	Correlational	Data obtained from Centers for Disease control/National HIV Surveillance System (NHSS) and U.S. Census Bureau's American Community Survey (ACS) Data from 2018	100%	HIV, mortality risk, social determinants of health, Black women, county level, poverty, employment, health insurance	Ecological analysis found poverty and lack of health insurance to be predictors of mortality, suggesting a need for increased prevention, care, and policy efforts targeting Black women with HIV who live in environments characterized by increased poverty and lack of health insurance

BWLH, Black women living with HIV.

## Results

**FIG. 1.**
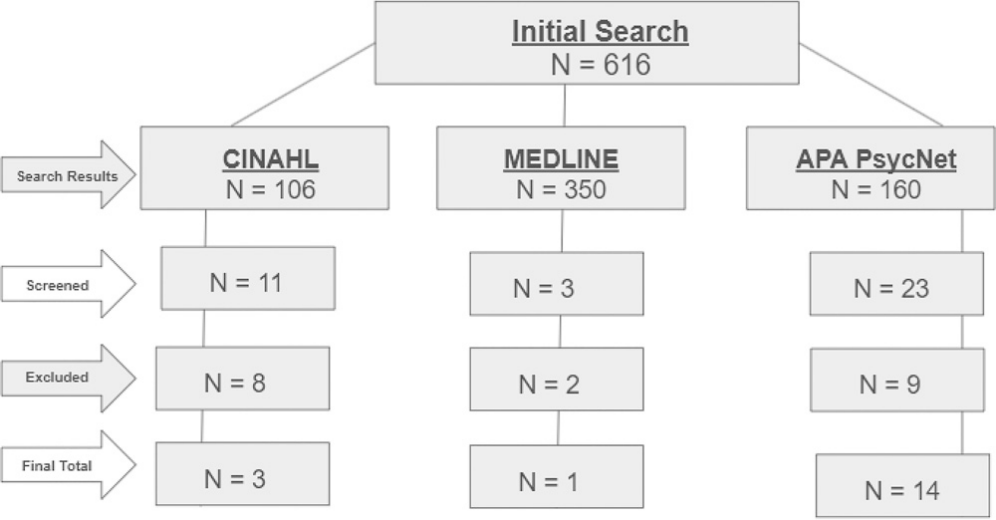
Graphic of the extraction method for generating the final sample of articles. The following figure, beginning with the total amount of articles that appeared in the search, demonstrates each step in the article screening process. The final sample of 18 articles that fit the screening criteria can be seen in the last row.

From the screening process detailed in the proceeding section, 37 articles that fit the initial qualifications for inclusion were identified across the databases. The articles extracted from each database featured 11 from CINAHL, 3 from MEDLINE, and 23 from APA PsycNet. Upon further screening beyond the title and abstract, 20 articles were excluded from the final sample for not focusing on the target population of Black, cisgender, adult women living with HIV, failed to discuss health disparities in the abstract, or were found to be a duplicate of an article identified previously from another database. Specifically, from the 20 not included in this scoping review, 8 were from CINAHL, 2 were from MEDLINE, and 10 were from APAPsycNet. Of these 20 excluded studies, one article from MEDLINE and one from APA PsycNet were found to be a duplicate of another article already represented in the review. One article from APA PsycNet met all other qualifications but was excluded because Black women only comprised 32% of the sample. In the end, 18 articles comprised the final sample, including three from CINAHL, one from MEDLINE, and 14 from APA PsycNet (Fig. 1).

Within the final sample of 18 studies, 14 focused on quantitative studies, while only four featured findings from qualitative studies. The average percentage of Black women within study samples was 84%, where BWLH ranged from 61% to 100%. All studies were conducted in the United States per the inclusion requirements. Furthermore, most studies highlighted subject data collected in the Southeast United States. Exceptions to this included participant data gathered from cities including Brooklyn, NY; Bronx, NY; Chicago, IL; Boston, MA; and San Francisco, CA.

Five leading themes emerged from the literature: discrimination and stigma, poverty, mental and physical health, health care, and social support. Analyzing the findings within these five areas played influential roles, either positive or negative, in BWLH progression along the HIV care continuum.

### Discrimination and stigma

BWLH are impacted by discrimination and stigma that affect their access and engagement in HIV care and management of other chronic health problems, according to several studies.^10–17^ BWLH’s inability to disclose their HIV status when seeking health care and not keeping their appointments are associated with perceived discrimination and stigma.^12,13^ According to one study, there is an association between intersectional discrimination and microaggression with both depression and post-traumatic stress disorder (PTSD). Intersectional discrimination refers to the presence of multiple oppressive factors (e.g., lack of access to health care, denial of housing, neighborhoods devoid of opportunity), resulting in systems of oppression impacting minority groups’ quality of life. In comparison, microaggressions are subtle forms of discrimination experienced every day (e.g., daily subtle insults, jokes). Existing theories, including Microaggression theory, relate discrimination, and microaggressions to negative health outcomes.^10^ Among BWLH, internalized HIV stigma, internalization, and self-rejection are associated with their perceived chances of being diagnosed with breast cancer and whether they would participate in breast cancer or other health screening activities.^18,19^ Another study indicated that HIV-related stigma demonstrated in health care settings was associated with a lower level of trust in providers.^20^ Providers can make a positive impact to help reduce internalized HIV-related stigma, through assessment of BWLH knowledge of the virus, demolishing any misconceptions of the disease that possibly result in BWLH disclosing HIV status to support systems.^17^ One study did not observe an association between internalized and enacted HIV-related stigma and lower engagement in HIV care among BWLH. Enacted HIV stigma is defined as the experiences of discrimination, stereotyping, or prejudice from others because of one’s HIV status.^19^ However, other factors related to consistent engagement in HIV care were advanced age, higher educational attainment, a strong social support system, and the type of HIV clinic setting where health care was received.^16^

### Poverty

Studies found a correlation between neighborhood poverty, the use of ART and HIV viral loads, and mortality.^13,21,22^ Neighborhood poverty plays a significant role in higher prevalences of unsuppressed viral loads, and a decrease in the proportion of BWLH receiving ART.^13,21,22^ Areas with a high level of neighborhood poverty can be associated with structural factors, such as the lack of HIV services at clinics that serve low-income individuals.^22^ In addition to having an HIV-positive status, BWLH’s poor socioeconomic status contributed to the inability to afford stable housing or sustain employment.^13^ Women 18–54 years of age living in counties with lower poverty levels and who were insured had lower mortality rates.^23^ Few studies have examined U.S.-born Black women and non-U.S.-born women. In this review, one study assessed disparities linked with social and economic disadvantages to diagnosing HIV, and the results identified a relative disparity in HIV diagnosis rates for non-U.S. Caribbean and African-born women, an increase of 25%.^24^ The second study using the Social Ecological Framework indicates that individual, community, and societal factors can contribute to a person’s overall health. Non-U.S.-born women were most likely to have a higher viral suppression rate in comparison to U.S.-born women when education or housing status were not contributing factors.^25^

### Mental and physical health

Having HIV, along with mental health and physical health problems, deterred some women from being actively involved in their HIV care and treatment.^10,13^ However, a study reported that some women became engaged in their HIV care when diagnosed with other comorbidities.^13^ Another study found that mental health was impacted negatively by microaggressions associated with racism, sexism, heterosexism, and HIV-related stigma when experienced by BWLH.^10^

Two studies looked at the association between HIV and breast health.^18,19^ There was an association between symptoms of depression and HIV-related stigma and perception of being diagnosed with breast cancer.^19^ Findings of a study examining the association of internalized HIV-related stigma and beliefs about breast health found HIV-related stigma correlated with one’s inclination to have breast cancer and possible undesirable effects of breast cancer.^18^ One study found high psychosocial stress positively related to increased neuroendocrine biomarker concentrations among BWLH, suggesting the association with cardiovascular disease.^26^

### Health care

Studies recognized several factors within health care affecting BWLHs linked to HIV care.^12–14,20,22,27^ Structural factors in high-poverty neighborhoods, such as insufficient health care providers or access to ART in underserved clinics, contribute to health disparity.^23^ BWLH have avoided clinics in their community or where people they know would recognize them, which deterred them from receiving HIV care.^13^ Their willingness to be engaged in HIV care occurred when they felt appreciated by health care providers who listened to their stories and knew they were at the center of their health care.^12,14,20^ BWLH, who had a high trust level in health care providers, correlated with higher odds of adherence to ART, and was likelier to trust the health care system.^12,27^

### Social support

Studies found that social support helps with ART adherence, effectively coping with psychosocial issues, and building resilience.^10,12–14,20^ BWLH families and healthy intimate relationships are essential for building resiliency.^12,13^ Support from other women living with HIV was most helpful when first diagnosed in helping them learn how to adjust to their diagnosis.^12,14^ Support from peers helped BWLH when faced with enacted HIV-related stigma and discrimination in addition to race-related discrimination.^12^ Having social support where they received their HIV care services helped them resist feeling ashamed of living with the disease and have self-compassion.^20^ Support received from health care providers helped women cope with other health conditions not related to HIV diagnosis.^12,20^

## Discussion

People with HIV-positive status can live longer with ART, but they still encounter barriers to achieving and maintaining viral suppression.^4^ This article examines the disparities and inequities BWLH experience in HIV care and treatment management. Based on the inclusion and exclusion criteria, the review identified 18 U.S.-based studies with four main themes related to HIV care: discrimination, poverty, mental and physical health, health care, and social support. The findings increase understanding of factors that hinder HIV care continuum engagement among BWLH, contributing to suboptimal outcomes.

Studies have examined the disparities experienced by BWLH due to racial and HIV discrimination and stigma, as well as the role of gendered microaggressions and intersectional microaggressions. Inequities in health are rooted in stigma.^17^ BWLH’s stigma may be internalized, anticipated, or enacted. Fear of developing a disease other than HIV is attributed to internalized HIV-related stigma. Other individuals’ discrimination or prejudice, such as HIV-related enacted stigma experiences, did not inhibit their participation in health promotion activities. Another study found no association between continuous engagement in HIV care and internalized or enacted stigma. However, BWLH have concerns of being stigmatized (i.e., misconceptions) for having an HIV diagnosis. This is associated with not disclosing their HIV status when seeking health care. If BWLH detects HIV-related stigma among their community members, it discourages participation in HIV care, increases the likelihood of missed visits, and reduces trust in health care providers.^3,16,17,19,20^

A BWLH may experience microaggressions such as daily subtle insults, jokes, or unintentional comments related to their HIV status that make them feel ashamed of their HIV status. In addition to gendered microaggressions, Black women also experience intersectional microaggressions as they are both women and Black. The implications of gendered racial microaggressions on barriers to care are greater than those of race-related or HIV-related discrimination.^10,11,21^ In microaggression theory, physical and mental health can be adversely affected by microaggression.^28^ Several studies have linked microaggressions to mental health issues among BWLH. Moreover, HIV-related discrimination, racial discrimination, and gendered racial microaggressions are associated with structural and institutional oppression. BWLH are at risk of structural and institutional oppression when experiencing health care access limitations, inadequacies in transportation infrastructure, and medical providers deficient in HIV care knowledge. Social support helps BWLH enhance their coping mechanisms to avoid stress or unpleasant emotions when faced with stigma and discrimination.^10,11,21^

A robust social support system plays a crucial role in BWLH’s engagement in HIV care. In this review, several factors were identified that contributed to BWLH not seeking HIV care once diagnosed, not starting ART, not adhering to ART, not being retained in HIV care, and, ultimately, the inability to maintain viral suppression. A woman’s perception of discrimination or the internal stigma of feeling ashamed or anticipating stigma when entering the health care system are contributing reasons to deterring care. Moreover, a breach of patient confidentiality is another concern when entering the health care system. As a result, a strong social support system helps overcome potential barriers to HIV care. Furthermore, health care organizations should provide patient-centered and team-oriented services.

BWLH living in poverty may not understand the significance of physical and mental health and how to navigate the health care system when structural factors exist. BWLH may be uninformed about HIV disease progression, the benefits of being connected to HIV care, and how HIV can be managed like other chronic health conditions. In addition, BWLH may not be aware of the resources available to assist with the cost of services when they are first diagnosed with HIV. Low-income individuals may be able to retain care with the help of community services. Women may not have access to transportation services to obtain HIV care outside of their neighborhoods, where they may prefer to receive treatment to avoid being recognized. For BWLH, it is essential to receive care for non-HIV-related health conditions, to participate in health promotion, and to be screened for diseases before they develop. The mental health status of BWLH plays a crucial role in their commitment to engage in HIV care to decrease the health disparities that are associated with being Black and HIV-positive. Access to health care should be available to BWLH where HIV-related stigma is not prevalent, the ability to receive ART, and the availability of HIV care management services (e.g., case management, behavioral health, mental health, dental), regardless of income level.

This study’s limitations include limiting the search to three databases, which may have prevented studies from being considered in the review. Many of the studies consisted of states in the southeast without a representative sample of other regions in the United States. A limited number of interventions were considered in the study findings to increase BWLH participation in HIV care. Future studies should explore the impact of gender and racial microaggressions on BWLH’s initiation of HIV care when first diagnosed with HIV and throughout their health care management. Moreover, future research efforts could investigate the effectiveness of policies and procedures adopted by health care institutions to increase HIV retention rates and viral suppression rates among BWLH.

## Conclusion

This literature review contributes to a deeper understanding of HIV-related health factors that impact Black women in the United States. Various challenges were posed by race and ethnicity, socioeconomic status, and individual situations. Compared to women of other races and ethnicities, Black women suffer from a higher rate of HIV diagnosis. The existence of these disparities may be influenced by socioeconomic factors such as poverty, social determinants of health, the lack of access to health care, stigma, and discrimination. A woman’s ability to protect herself can be impacted by individual circumstances such as gender inequality and power dynamics that affect relationships, neglect toward HIV prevention and treatment, and a lack of awareness and education that hinders early diagnosis and intervention. Adherence to medication and overall health can be affected by physical and mental health conditions, as well as substance use.

An integrated and multifaceted approach is needed to address the complex challenges that Black women with HIV face. The promotion of education and awareness is an effective preventative strategy for HIV that empowers individuals. Second, reducing stigma and discrimination related to HIV and creating a communicative environment is essential to encouraging testing and treatment. In addition, affordable medications and easy access to health care can make services more effective. To provide comprehensive services to Black women with HIV, health care professionals, and community organizations could collaborate holistically and culturally.

## Abbreviations Used

ART: Antiretroviral Therapy
BWLH: Black Women Living with HIV
PTSD: Post-traumatic Stress Syndrome

## References

[1] HIV.gov. HIV & AIDS Trends and U.S. Statistics Overview. Available from: https://www.hiv.gov/hiv-basics/overview/data-and-trends/statistics [Last accessed: January 11, 2024].

[2] HIV.gov. HIV Statistics Impact on Racial and Ethnic Minorities. Available from: https://www.hiv.gov/hiv-basics/overview/data-and-trends/impact-on-racial-and-ethnic-minorities [Last accessed: January 11, 2024].

[3] Centers for Disease Control and Prevention (CDC). 2022. HIV and Women: HIV Diagnoses. Available from: https://www.cdc.gov/hiv/group/gender/women/diagnoses.html [Last accessed: January 11, 2024].

[4] Prevention Challenges | HIV and African American People | Race/Ethnicity | HIV by Group | HIV/AIDS. CDC. 2023. Available from: https://www.cdc.gov/hiv/group/racialethnic/africanamericans/prevention-challenges.html [Last accessed: December 6, 2023].

[5] Morse GD, Nanzigu S. Frontiers in HIV research. In: Advances in HIV Treatment: HIV Enzyme Inhibitors and Antiretroviral Therapy. Volume 1. Bentham Science Publisher: Sharjah, UAE; 2015; P 1.

[6] Centers for Disease Control and Prevention (CDC). 2022. Pre-Exposure Prophylaxis (PrEP). Available from: https://www.cdc.gov/hiv/risk/prep/index.html [Last accessed: March 10, 2024].

[7] Centers for Disease Control and Prevention (CDC). 2022. Post-Exposure Prophylaxis (PEP). Post-Exposure Prophylaxis (PEP) | HIV Risk and Prevention | HIV/AIDS | CDC.

[8] Kay ES, Batey DS, Mugavero MJ. The HIV treatment cascade and care continuum: Updates, goals, and recommendations for the future. AIDS Res Ther 2016;13(1):35; doi: 10.1186/s12981-016-0120-0 27826353 PMC5100316

[9] Arksey H, O’Malley L. Scoping studies: Towards a methodological framework. Int J Soc Res Methodol 2005;8(1):19–32; doi: 10.1080/1364557032000119616

[10] Dale SK, Nelson CM, Wright IA, et al. Structural equation model of intersectional microaggressions, discrimination, resilience, and mental health among black women with HIV. Health Psychol 2023;42(5):299–313; doi: 10.1037/hea0001275 37141016 PMC10167554

[11] Dale SK, Dean T, Sharma R, et al. Microaggressions and discrimination relate to barriers to care among black women living with HIV. AIDS Patient Care STDS 2019;33(4):175–183; doi: 10.1089/apc.2018.0258 30932695 PMC6459277

[12] Dale SK, Safren SA. Resilience takes a village: Black women utilize support from their community to foster resilience against multiple adversities. AIDS Care 2018;30(sup5):S18–S26; doi: 10.1080/09540121.2018.1503225 30628458 PMC6430675

[13] Ojukwu E, Cianelli R, Villegas Rodriguez N, et al. A qualitative study on the social determinants of HIV treatment engagement among black older women living with HIV in the southeastern United States. J Assoc Nurses AIDS Care 2022;33(2):211–223; doi: 10.1097/JNC.0000000000000299 35195612

[14] Koch A, Ritchwood TD, Bailey DE, et al. Exploring resilience among black women living with HIV in the southern United States: Findings from a qualitative Study. J Assoc Nurses AIDS Care 2022;33(2):224–234; doi: 10.1097/JNC.0000000000000311 35195613 PMC9188835

[15] Cressman AE, Howe CJ, Nunn AS, et al. The relationship between discrimination and missed HIV care appointments among women living with HIV. AIDS Behav 2020;24(1):151–164; doi: 10.1007/s10461-019-02522-81 31049811 PMC6824941

[16] McCoy K, Lipira L, Kemp CG, et al. Exploring HIV-related stigma as a determinant of engagement in HIV care by African American women. J Assoc Nurses AIDS Care 2020;31(2):167–175; doi: 10.1097/JNC.0000000000000140 31725104 PMC7093210

[17] McMillian-Bohler JM, Holt L, Adimora AA, et al. Examining stigma and disclosure among women with HIV in the southern United States: Qualitative study guided by the adaptive leadership framework for chronic illness. J Assoc Nurses AIDS Care 2023;34(1):113–124; doi: 10.1097/JNC.0000000000000354 35862630 PMC10122520

[18] Fabian K, Molina Y, Kemp CG, et al. Internalized HIV-related stigma and breast health beliefs among African American women receiving care for HIV in the USA. J Racial Ethn Health Disparities 2020;7(1):45–51; doi: 10.1007/s40615-019-00632-6 31452148 PMC6980483

[19] Molina Y, Scheel JR, Endeshaw M, et al. Enacted HIV-related stigma and breast-health beliefs and practices among African American women living with HIV: The mediating roles of internalized HIV-related stigma and depressive symptoms. Stigma Health 2018;3(4):377–384; doi: 10.1037/sah0000105 30525110 PMC6277041

[20] Budhwani H, Yigit I, Ofotokun I, et al. Examining the relationships between experienced and anticipated stigma in health care settings, patient–provider race concordance, and trust in providers among women living with HIV. AIDS Patient Care STDS 2021;35(11):441–448; doi: 10.1089/apc.2021.0096 34739336 PMC8817693

[21] Raiford JL, Yuan X, Carree T, et al. Understanding disparities in antiretroviral therapy adherence and sustained viral suppression among black, Hispanic/Latina, and white women in the United States – medical monitoring project, United States, 2015–2019. J Acquir Immune Defic Syndr 2023;93(5):413–421; doi: 10.1097/QAI.0000000000003214 37129907 PMC10524626

[22] Cope AB, Edmonds A, Ludema C, et al. Neighborhood poverty and control of HIV, hypertension, and diabetes in the women’s interagency HIV study. AIDS Behav 2020;24(7):2033–2044; doi: 10.1007/s10461-019-02757-5 31907676 PMC7319872

[23] Watson L, Gant Z, Hu X, et al. Exploring social determinants of health as predictors of mortality during 2012–2016, among black women with diagnosed HIV infection attributed to heterosexual contact, United States. J Racial Ethn Health Disparities 2019;6(5):892–899; doi: 10.1007/s40615-019-00589-6 30980295

[24] Demeke HB, Johnson AS, Wu B, et al. Unequal declines in absolute and relative disparities in HIV diagnoses among black women, United States, 2008 to 2016. Am J Public Health 2018;108(S4):S299–S303; doi: 10.2105/AJPH.2018.304641 30383429 PMC6215380

[25] Nace A, Johnson G, Eastwood E. Comparison of HIV viral suppression between a sample of foreign-born and U.S.-born women of color in the United States. J Immigr Minor Health 2021;23(6):1129–1135; doi: 10.1007/s10903-021-01213-8 33974177

[26] Levy ME, Waters A, Sen S, et al. Psychosocial stress and neuroendocrine biomarker concentrations among women living with or without HIV. PLoS One 2021;16(12):e0261746; doi: 10.1371/journal.pone.0261746 34941922 PMC8699620

[27] Michel KG, Ocampo JMF, Spence AB, et al. High provider trust associates with high HIV antiretroviral adherence among women living with HIV in a metropolitan Washington, DC cohort. AIDS Patient Care STDS 2022;36(1):17–25; doi: 10.1089/apc.2021.0110 34910888 PMC8905303

[28] Nadal KL, Griffin KE, Wong Y, et al. The impact of racial microaggressions on mental health: Counseling implications for clients of color. J Counseling Develop 2014;92(1):57–66; doi: 10.1002/j.1556-6676.2014.00130.x

